# Dysphasische Anfälle infolge einer chronischen Leptomeningitis: Eine differenzialdiagnostische Herausforderung

**DOI:** 10.1007/s00115-021-01190-1

**Published:** 2021-09-29

**Authors:** K. Olaciregui Dague, J. Pukropski, C. Hummel, A. Becker, R. Surges, T. Baumgartner

**Affiliations:** 1 Klinik und Poliklinik für Epileptologie, Universitätsklinikum Bonn, Venusberg-Campus 1, 53127 Bonn, Deutschland; 2 Institut für Neuropathologie, Sektion für Translationale Epilepsieforschung, Universitätsklinikum Bonn, Bonn, Deutschland

## Fallbeschreibung

Die 80-jährige Patientin stellte sich zur diagnostischen Zuordnung rezidivierender Episoden mit Sprachstörung in unserer Klinik vor. Erstmalig sei es vor ca. 6 Monaten zu einer Episode mit Wortfindungs- und Sprachverständnisstörungen für wenige Minuten gekommen. Die erste Episode sei von Parästhesien im rechten Arm begleitet gewesen. Daraufhin erfolgte eine Aufnahme in einer auswärtigen Klinik unter dem Verdacht einer transitorischen ischämischen Attacke. In der Magnetresonanztomographie (MRT) des Neurokraniums fand sich kein Hinweis auf eine frische Ischämie. Es zeigte sich jedoch eine kontrastmittelaufnehmende Verdickung der Leptomeninx links temporookzipital. In der daraufhin durchgeführten Liquordiagnostik fanden sich zunächst unauffällige Parameter (Zellzahl, Glukose, Eiweiß und Laktat in der Norm). Eine intrathekale Immunglobulinsynthese war nicht nachweisbar. Die Erregerdiagnostik zeigte ebenfalls keine Auffälligkeiten im Liquor (Borrelien, neurotrope Viren: HSV1/2, Masern, Röteln, VZV). In der Röntgenaufnahme des Thorax fand sich neben dem bekannten Situs inversus eine ovaläre Verschattung parahilär links mit einem Durchmesser von ca. 15 mm. Die Patientin wurde zunächst mit ASS 100 mg/Tag in der Annahme einer transitorischen ischämischen Attacke entlassen. Etwa 5 Monate später ereigneten sich zwei weitere Episoden mit Sprachstörung, weswegen die Patientin erneut in derselben Klinik vorstellig wurde. Magnetresonanztomographisch fand sich eine zunehmende Ausdehnung der kontrastaufnehmenden Verdickung der Leptomeninx linkshemisphärisch. Liquorchemisch war eine geringe Pleozytose (6 Leukozyten/µl; Normwert: <5/µl) und eine geringe Eiweißerhöhung (501 mg/l; Normwert 150–450 mg/l) nachweisbar. Im Reiber-Schema bestand eine intrathekale IgA- und IgM-Antikörpersynthese. Tumorzellen fanden sich im Liquor nicht. Unter der Annahme rezidivierender fokaler Anfälle wurde eine antikonvulsive Therapie mit Levetiracetam etabliert.

Zur Vorgeschichte ist zu berichten, dass die Patientin an einer seropositiven (Rheumafaktor und Anti-CCP-Antikörper) rheumatoiden Arthritis leidet (Erstdiagnose 2000). Es wurde zunächst eine Basistherapie mit Methotrexat etabliert, die aufgrund von Blutbildveränderungen auf Leflunomid umgestellt wurde. Bei unzureichender Krankheitskontrolle wurde die Basistherapie im Jahr 2015 auf Certolizumab umgestellt, welche in Kombination mit Prednisolon 2,5 mg/die bis zur Aufnahme in unserer Klink fortgeführt wurde.

**Figure 1:**
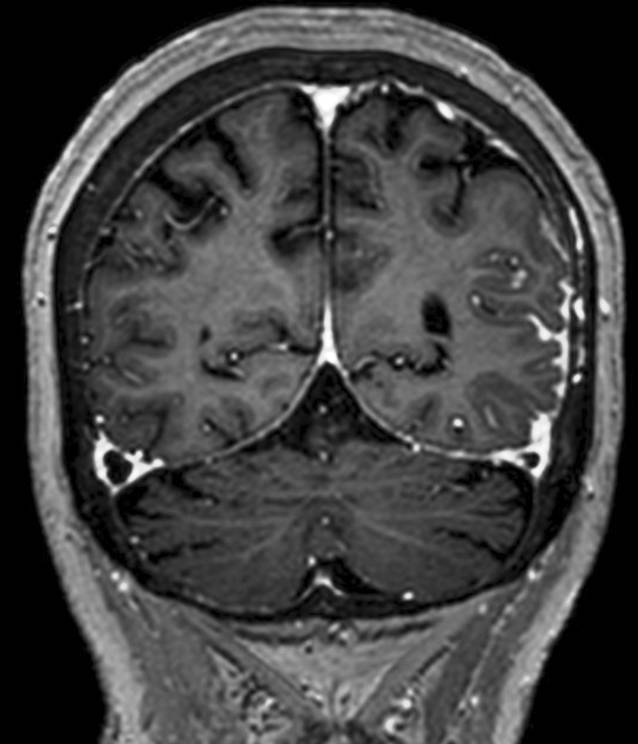

**Figure 2:**
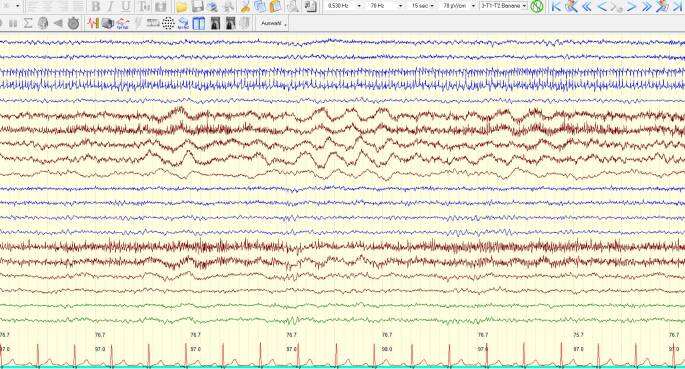

**Figure 3:**
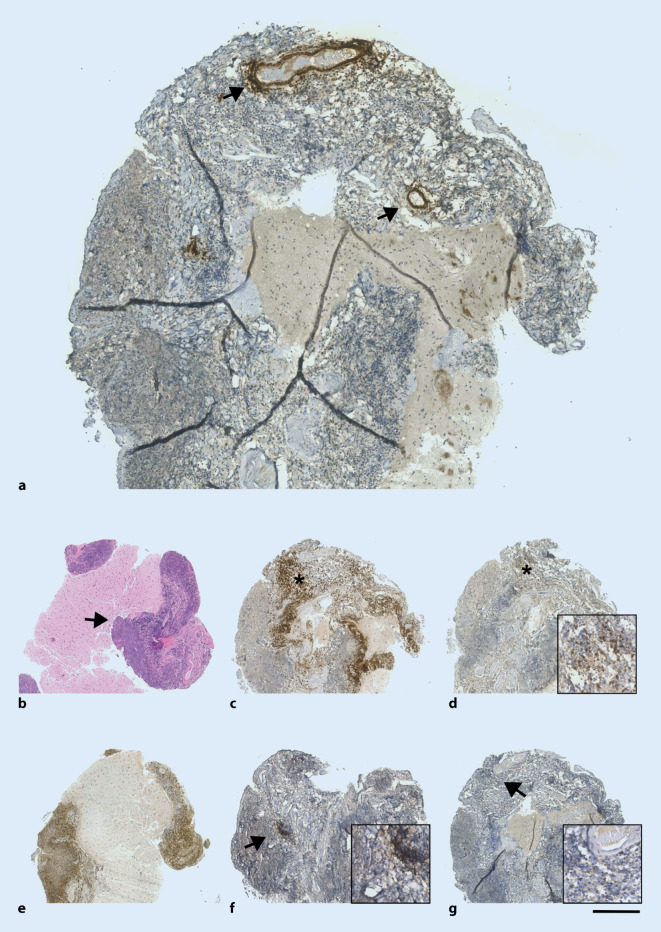

Bei der ersten Vorstellung in unserer Klinik zeigte die Patientin bis auf leichte Wortfindungsstörungen kein fokal-neurologisches Defizit. Unter einer Therapie mit Levetiracetam hatten sich in der Zwischenzeit zwei weitere Episoden mit Sprachstörung ereignet. In der MRT des Neurokraniums stellte sich ein nahezu konstantes Bild der leptomeningeal betonten Kontrastmittelanreicherung dar (Abb. 1). Bildgebende Hinweise auf eine zerebrale Amyloidangiopathie zeigten sich nicht. Eine ergänzende MRT des Myelons erbrachte keine Auffälligkeiten. In der Video-EEG-Ableitung zeigte sich ein kontinuierlicher Theta-Delta-Verlangsamungsherd links temporookzipital (Abb. 2). Liquorchemisch fanden sich eine intrathekale Drei-Klassen-Synthese (IgG, IgA, IgM) und eine lymphozytäre Pleozytose von 24 Zellen/µl (Normwert: <5/µl). Eine erneute umfangreiche virologische und mikrobiologische Erregerdiagnostik in Serum und Liquor mit Fokus auf atypische Erreger blieb ergebnislos (Mykobakterien, Borrelien, Spirochäten, *Cryptococcus neoformans, Brucella spp., Nocardia spp.*). Neuropathologisch gelang weiterhin kein Tumorzellnachweis im Liquor. Rheumafaktor (129,7 IU/ml, Normwert <14 IU/ml) und Anti-CCP-Antikörper (>2500 U/ml, Normwert <17 U/ml) waren deutlich erhöht. C‑reaktives Protein, Angiotensin Converting Enzyme, Interleukin-2-Rezeptor, antinukleäre Antikörper sowie zytoplasmatische und perinukleäre antineutrophile zytoplasmatische Antikörper waren im Normbereich. Eine weiterführende Diagnostik mittels Computertomographie (CT) des Thorax erbrachte mehrere partiell verkalkte Rundherde in beiden Lungenfeldern mit einer maximalen Größe von 11 × 15 mm. Eine Biopsie eines Lungenrundherdes mittels Bronchoskopie gelang nicht. Die mikrobiologischen Befunde der bronchoalveolären Lavage erbrachten keine Auffälligkeiten. Aufgrund einer Zunahme der leptomeningealen Kontrastmittelanreicherung 2 Wochen später erfolgte die Durchführung einer Hirnbiopsie links parietal. Im neuropathologischen Bericht zeigte sich das Bild einer zerebralen Amyloidangiopathie mit Nachweis leptomengingeal akzentuiert imponierender, floride-entzündlicher Infiltrate (Abb. 3). Ein Erreger- oder Tumornachweis erfolgte auch in der Gehirnbiopsie nicht.

## Therapie und Verlauf

Nach Vorstellung der Patientin in unserer Klinik wurde zunächst die antikonvulsive Therapie um Lacosamid erweitert. Zudem wurde die Therapie mit Certolizumab pausiert. In den darauffolgenden 3 Wochen wurden keine Anfälle mehr beobachtet. Jedoch zeigte sich die Sprachstörung fortschreitend, und die Patientin entwickelte ein leichtes Delir. Aufgrund des fortschreitenden klinischen und radiologischen Befundes erfolgte auch bei fehlendem Erregernachweis eine kalkulierte antibiotische Therapie mit Vancomycin und Meropenem. Hierunter kam es jedoch zu keiner Besserung des klinischen Befundes, so dass die Therapie bei ebenfalls fehlendem Erregernachweis in der Hirngewebsdiagnostik beendet wurde.

**Figure 4:**
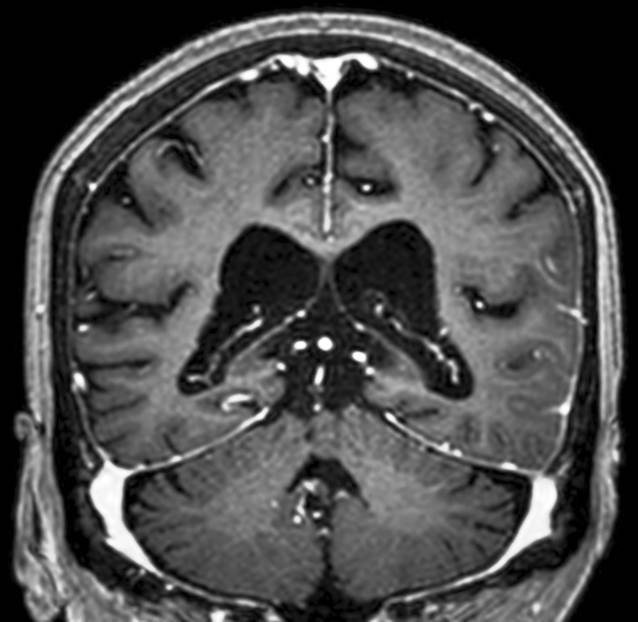

Etwa 6 Wochen nach der ersten Vorstellung in unserer Klinik erfolgte eine Kortisonstoßtherapie über 3 Tage (Methylprednisolon 500 mg/Tag) und eine anschließende orale Kortisontherapie (Prednisolon 1 mg/kg/KG). Bereits eine Woche nach Beginn der Kortisontherapie kam es zu einer deutlichen Befundbesserung in einer zerebralen Kernspintomographie (Abb. 4). Zwei Monate später war keine leptomeningeale Kontrastmittelaufnahme mehr nachweisbar, der Liquor zeigte normwertige Befunde, und Episoden mit Sprachstörung traten nicht mehr auf.

## Diskussion

Der vorgestellte Fall demonstriert das breite differenzialdiagnostische Spektrum einer chronischen Leptomeningitis (Tab. 1).

**Table 1:** 

Chronische Meningitis	
**Septisch**	*Bakterien:* Myokobakterien (insbesondere *Mycobacterium tuberculosis*), Nokardien, Brucellose, *Treponema pallidum, Borrelia burgdorferi*
	*Pilze:* insbesondere *Cryptococcus neoformans, Aspergillus, Candida*
	*Parasiten: Toxoplasma gondii*
	*Viren:* Enteroviren, HSV 1 und 2, HHV 6, VZV
**Aseptisch**	**Meningeosis carcinomatosa**
	*Autoimmun-rheumatische Erkrankungen:* rheumathoide Meningitis, systemischer Lupus erythematodes, Morbus Behçet
	*Weitere immunologische Erkrankungen:* Neurosarkoidose, primäre ZNS-Vaskulitis, ZNS-Beteiligung bei systemischer Vaskulitis
	*Medikamentös induziert:* IVIGs, Monoklonale Antikörper, nichtsteroidale Antirheumatika, Methotrexat
	*Andere Ursachen:* zerebrale Amyloidangiopathie assoziiert mit Inflammation

Im Fall unserer Patientin wurde aufgrund der langjährigen immunsuppressiven Therapie und des zunächst subakuten Krankheitsprogresses insbesondere eine atypische, erregerbedingte Ursache in Betracht gezogen. Unter Berücksichtigung der unklaren Lungenrundherde wurden neben dem Vorliegen eines Bronchialkarzinoms oder einer tuberkulösen Meningitis auch seltene opportunistische Erreger wie u. a. Nokardien diskutiert. Insbesondere die Diagnosestellung einer tuberkulösen Meningitis kann Behandler vor große Schwierigkeiten stellen, da sowohl die PCR aus dem Liquor (Sensitivität: 50–60 %) als auch der mikroskopische Nachweis von säurefesten Stäbchen (Sensitivität: 10–15 %) eine geringe bis mäßige Sensitivität zeigen. Der kulturelle Nachweis dauert zudem mehrere Wochen [1]. Weitere Erreger (insbesondere Mykosen, Brucellose, neurotrope Viren) mussten ebenfalls differenzialdiagnostisch berücksichtigt werden. Hinweise auf eine Neurosarkoidose fanden sich laborchemisch nicht.

Zudem wurde eine aseptische Meningitis infolge der TNF-Blocker-Therapie mit Certolizumab diskutiert. Mehrere Einzelfallberichte einer aseptischen Meningitis unter verschiedenen TNF-Blockern sind in der Literatur beschrieben [3, 5]. Neben den o. g. Differenzialdiagnosen wurde eine rheumatoide Meningitis favorisiert. Die zum Zeitpunkt des Erkrankungsbeginns fehlende Krankheitsaktivität der rheumathoiden Arthritis andere Organsysteme betreffend und das Fehlen systemischer Entzündungszeichen, ließ jedoch zunächst an der Diagnose zweifeln.

Letztendlich wurde sich aufgrund der fortschreitenden Klinik insbesondere zum Ausschluss 1) einer fokalen Meningeosis carcinomatosa (auch bei mehrfach fehlendem Tumornachweis im Liquor), 2) einer zerebralen Vaskulitis und 3) einer tuberkulösen Meningitis für eine Hirnbiopsie entschieden. Der neuropathologische Befund erbrachte ein Bild, welches mit einer zerebralen Amyloidangiopathie assoziiert mit Inflammation („cerebral amyloid angiopathy-related inflammation“, CAA-RI) vereinbar war. Auch wenn die zerebrale MRT (kein Nachweis einer superfiziellen Siderose, nur einzelne Microbleeds) nicht das typische Bild einer zerebralen Amyloidangiopathie zeigte, war eine CAA-RI mit initialer leptomeningealer Affektion nicht ausgeschlossen (s. auch [2]).

Nach bestmöglichem Ausschluss einer infektiös erregerbedingten Genese wurde sich für eine Kortisonstoßtherapie in der Annahme einer rheumatoiden Meningitis entschieden. Das schnelle und sehr gute Ansprechen unterstützte klar diese Diagnose. Den histologischen Nachweis der zerebralen Amyloidangiopathie interpretieren wir rückblickend als koinzidenten Befund einer in dieser Altersgruppe nicht seltenen Pathologie [4] ohne sicheren Zusammenhang mit dem aktuellen Krankheitsgeschehen.
